# Use of a Six-Item Modified Frailty Index to Predict 30-day Adverse Events, Readmission, and Mortality in Older Patients Undergoing Surgical Fixation of Lower Extremity, Pelvic, and Acetabular Fractures

**DOI:** 10.5435/JAAOSGlobal-D-22-00286

**Published:** 2023-01-19

**Authors:** Christian A. Pean, Hannah M. Thomas, Upender M. Singh, Malcolm R. DeBaun, Michael J. Weaver, Arvind G. von Keudell

**Affiliations:** From the Duke Department of Orthopedic Trauma Surgery, University Health System, Durham, NC (Dr. Pean and Dr. DeBaun); the Harvard Orthopedic Trauma Initiative, Brigham and Women's Hospital/Massachusetts General Hospital, Boston, MA (Thomas, Dr. Weaver, and Dr. von Keudell); the Rigshospitalet, University of Copenhagen, Copenhagen, Denmark (Dr. Singh and Dr. von Keudell), and Bispebjerg Hospital, University of Copenhagen, Copenhagen, Denmark (Dr. von Keudell).

## Abstract

This is a retrospective study evaluating the use of a new six-item modified frailty index (MF-6) to predict short-term outcomes of patients receiving surgery for lower extremity fractures.

**Methods:**

Patients older than 65 years undergoing open reduction and internal fixation for lower extremity, pelvic, and acetabulum fractures were identified from the American College of Surgeons National Surgical Quality Improvement Program. The MF-6 was calculated by assigning one point for each of six common conditions. Multivariable analysis was used to compare patients with an MF-6 of <3 and ≥3. Outcome measures included complications, mortality, readmission, revision surgery, and length of stay. An area under the curve receiver operator analysis was conducted to compare the MF-6 with MF-5, an existing five-item frailty index.

**Results:**

Nine thousand four hundred sixty-three patients were included. Patients with an MF-6 of ≥3 were at markedly higher risk of discharge destination other than home (Exp[B] = 2.09), mortality (Exp[B] = 2.48), major adverse events (Exp[B] = 2.16), and readmission (Exp[B] = 1.82). Receiver-operating curve analysis demonstrated an area under the curve of 0.65 for mortality, 0.62 for major adverse events, and 0.62 for discharge destination other than home, all of which outperformed the MF-5.

**Discussion:**

The MF-6 was correlated with a 30-day postoperative incidence of infectious complications, readmission, and discharge destination. MF-6 scores can be used to risk-stratify patient populations as shifts to value-based care continue to develop.

Fractures of the lower extremities, pelvis, and acetabulum account for nearly one-third of fractures treated by orthopaedic surgeons,^1^ and they result in notable morbidity and mortality.^2^ The incidences of pelvic fractures, acetabular fractures, and almost all lower extremity fracture types seem to be increasing, likely driven by the aging population.^3,4,5,6,7^ Most hip and upper leg fractures are treated surgically, whereas around one-third of ankle fractures require internal fixation.^7,8,9^ Relatively few unstable pelvic ring and acetabulum fractures require surgical management (2% to 10% and 15%, respectively),^6,10^ although these fractures are commonly associated with additional injuries.^5,11^

As the population continues to age and the incidence of these fractures continues to increase, identification of patients at risk of postoperative complications and poor outcomes becomes increasingly important.^12,13,14^ Recent orthopaedic literature suggests that frailty, a measure of overall patient well-being, may be more useful than age or comorbidities alone when evaluating surgical risk among geriatric fracture patients.^15,16,17^ Saxton et al evaluated the relationship between frailty and complications among 226 general surgery patients and found that while age and number of comorbidities were not independently associated with increased risk of complications, there was an association between frailty and complications.^17^ In a study of 175 geriatric fracture patients, Gleason et al^15^ found that increased frailty measured using a short 5-item assessment conducted by geriatricians was associated with increased length of stay (LOS), postoperative complications, and discharge to a rehabilitation facility. Kojima et al^16^ conducted a systematic review of multiple frailty measures and found that increased frailty is a notable predictor of mortality.

Multiple models such as the Charlson Comorbidity Index, Elixhauser Comorbidity Measure, frailty phenotype, and frailty index have been used to categorize patients by risk level.^14,18,19,20^ In addition to these methods, a modified frailty index (MF-6), mapped to 11 common comorbidities that are recorded in the American College of Surgeons National Surgical Quality Improvement Program (ACS-NSQIP) database, predicts complications for elderly patients undergoing orthopaedic surgery.^21,22^ After 2012, the NSQIP required only five of these 11 variables to be reported mandatorily, resulting in the creation of a modified five-item frailty index (MF-5).^23^ Several studies have found the MF-5 to be a useful tool in identifying patients at risk of complications and poor outcomes after orthopaedic surgery.^13,24,25,26^

The five items used to estimate frailty in the MF-5 are diabetes mellitus, congestive heart failure, chronic obstructive pulmonary disease or current pneumonia, hypertension requiring medication, and nonindependent functional status.^13^ In addition to these variables, hypoalbuminemia is an important predictor of outcomes after orthopaedic surgery, although it is not routinely measured in clinical care nor included in frailty indices.^27,28,29,30,31^ In this study, hypoalbuminemia was considered in addition to the five items of the MF-5 to create a six-item modified frailty index (MF-6). The purpose of this study was to assess the use of the MF-6 in predicting short-term (≤30 days) adverse outcomes among patients older than 65 years undergoing open reduction and internal fixation for lower extremity, pelvic, and acetabular fractures in the inpatient setting.

## Methods

Using Current Procedural Terminology codes, patients aged 18 years and older with lower extremity, pelvic, and acetabular fractures undergoing surgical fixation between 2012 and 2019 were identified in the ACS-NSQIP database. ACS-NQSIP is a nationally validated, risk-adjusted, outcomes-based database currently used by over 700 hospitals. It has been used in in the orthopaedic literature for numerous large-scale studies and compared with similar databases such as the Nationwide Inpatient Sample with overall high fidelity. We selected pelvic, acetabular, and lower extremity fractures as the injuries of interest given the effect of these fractures on patient mobility, acuity, and their frequency among orthopaedic patients.

We narrowed patient selection to patients older than 65 years and who had complete perioperative data including albumin levels. The MF-6 consisted of a 6-point system with one point given to a patient for each of the six conditions on presentation for treatment: chronic obstructive pulmonary disease, congestive heart failure within 30 days before admission, insulin or non–insulin-dependent diabetes, hypertension requiring medication for treatment, partially dependent or fully dependent functional status, and hypoalbuminemia. Albumin levels less than 3.5 g/dL were categorized as hypoalbuminemia. Patients were categorized into two groups: those with a MF-6 of three or greater and those with an MF-6 of less than three.

Patient groups were compared for differences in preoperative comorbidities, adverse events, LOS, and readmissions in the 30-day postoperative period. The ACS-NSQIP tracks the occurrence of 23 individual adverse events during the first 30 postoperative days. “Major adverse event” was defined as the occurrence of any of the following: death, ventilation for more than 48 hours, unplanned intubation, stroke/cerebrovascular accident, pulmonary embolism, cardiac arrest, myocardial infarction, acute renal failure, sepsis, septic shock, or return to the operating room. We conducted an area under the curve (AUC) receiver operator analysis for the MF-6 compared with the MF-5 for major adverse events, readmission, mortality, and discharge disposition other than home. All statistical analyses were conducted using SPSS (IBM SPSS Statistics for Windows, Version 28.0: IBM Corp). Paired Student t-tests were used to assess continuous variables. Pearson chi square tests and odds ratios were used for categorical variables.

## Results

A total of 37,503 patients who underwent inpatient surgical fixation for lower extremity, pelvic, and acetabular fractures were identified in the database. On additional selection of patients older than 65 years and exclusion of patients with missing perioperative data, 9,463 patients were included in this study. A total of 26.7% of patients in the group had hypoalbuminemia. Patients with high MF-6 scores were on average older (78.1.3 years vs. 77.4 years, *P* < 0.01) and more likely to have American Society of Anesthesiologists status >2 (*P* < 0.01). When stratified by race, Black and Hispanic patients had significantly higher MF-6 scores on average compared with other patients (Table 1).

**Table 1:** Baseline Demographics and Univariate Analysis of Patient Characteristics Associated With MF-6 Greater Than Three

Patients (% total)	Normal MF-6 n = 6943 (73.4)	MF-6 > 3 n = 2520 (26.6)	Odds Ratio, Interfacility Transfer (95% Confidence Interval)
Sex			
Female	5,299 (76.3)	1,891 (75.0)	0.83 (0.84-1.04)
Age^a^	77.4 ± 8.0	78.1 ± 8.1	*P* < 0.001
BMI category			
<18	314 (4.5)	113 (4.5)	0.88 (0.80-1.24)
18-25^a^	2,126 (30.6)	550 (21.8)	0.63 (0.57-0.70)
25-30^a^	2,164 (31.2)	609 (24.2)	0.70 (0.63-0.78)
30-35	1,357 (19.5)	510 (20.2)	1.04 (0.93-1.17)
35-40^a^	588 (8.5)	327 (13.0)	1.61 (1.40-1.86)
>40^a^	394 (5.7)	411 (16.3)	3.24 (2.80-3.75)
Race^a^			
Hispanic^a^	395 (5.7)	225 (8.9)	1.63 (1.37-1.93)
Asian	126 (1.8)	49 (1.9)	1.07 (0.77-1.50)
Black^a^	331 (4.8)	172 (6.8)	1.46 (1.21-1.77)
White^a^	5,791 (83.4)	2,041 (81.0)	0.85 (0.75-0.95)
ASA>2	5,279 (76.0)	2,395 (95.0)	6.04 (5.00-7.29)
Fracture type			
Pelvis and acetabulum^a^	406 (5.8)	114 (4.5)	0.76 (0.62-0.94)
Femur^a^	3,332 (48.0)	1,415 (56.2)	1.39 (1.27-1.52)
Tibia and patella^a^	5,653 (81.4	1,290 (18.6)	0.74 (0.65-0.84)
Foot and ankle^a^	1,915 (27.6)	628 (24.9)	0.87 (0.79-0.97)
Diabetic^a^			
Yes	1,023 (14.7)	1,587 (63.0)	9.84 (8.87-10.93)
Bleeding disorder^a^	876 (12.6)	535 (21.2)	1.87 (1.66-2.10)
Smoker^a^			
Yes	683 (9.8)	287 (11.4)	11.18 (1.02-1.36)
Renal dialysis^a^			
Yes	112 (1.6)	139 (5.5)	3.56 (2.76-4.59)

ASA = American Society of Anesthesiologists, BMI = body mass index, CI = confidence interval, COPD = chronic obstructive pulmonary disease

a *P*<0.01, statistically significant.

**Figure 1:**
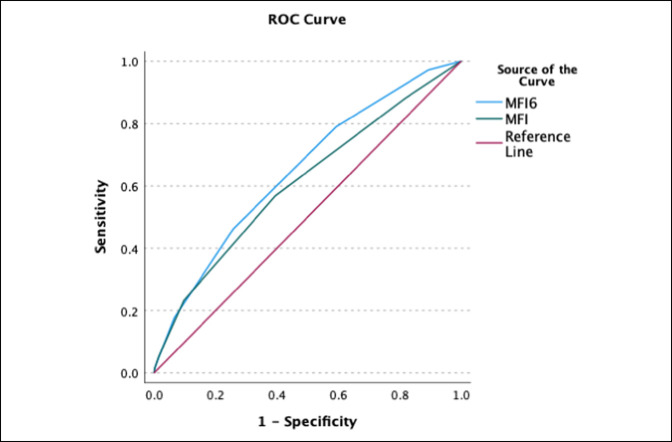
Graph showing receiver-operating curve of area under the curve (AUC) for MF-6 versus MF-5 for mortality. MF-6 AUC = 0.647, 95% CI 0.620 to 0.675; MF-5 AUC = 0.609, 95% CI 0.580 to 0.639.

**Figure 2:**
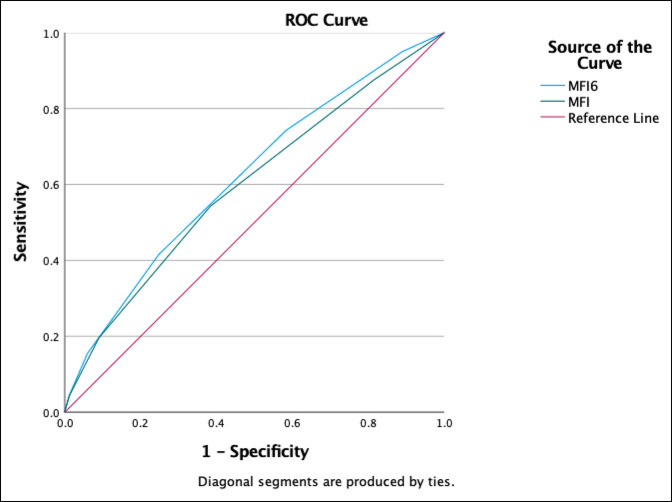
Graph showing receiver-operating curve of area under the curve (AUC) for MF-6 versus MF-5 for major adverse events. MF-6 AUC = 0.62, 95% CI 0.60 to 0.64; MF-5 AUC = 0.60, 95% CI 0.58 to 0.62.

**Figure 3:**
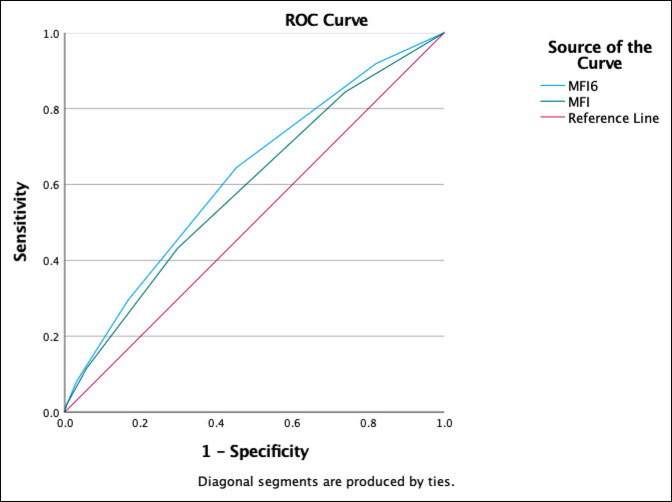
Graph showing receiver-operating curve of area under the curve (AUC) for MF-6 versus MF-5 for discharge disposition other than home. MF-6 AUC = 0.621, 95% CI 0.61 to 0.63; MF-5 AUC = 0.591, 95% CI 0.58 to 0.61.

Patients with high MF-6 scores had markedly higher rates of mortality (7.3% vs. 3.1%, OR 2.48, confidence interval [CI] 2.02 to 3.03), major adverse events (17.6% vs. 9.0% OR 2.16, CI 1.89 to 2.46), and infectious complications (10.7% vs. 6.6%, OR 1.7, CI 1.45 to 1.99). These patients also had a significantly higher readmission rate (11.6% vs. 6.8%, OR 1.82, CI 1.56 to 2.12, *P*<0.01), had longer LOS, and were more likely to be discharged to a nonhome location (Table 2). A receiver-operating curve linear regression analysis of the MF-6 demonstrated an AUC of 0.65 (CI 0.62 to 0.68) for mortality (Figure 1), AUC of 0.62 (CI 0.60 to 0.64) for major adverse events (Figure 2), and AUC of 0.62 (CI 0.61 to 0.63) for discharge destination other than home (Figure 3). The MF-6 out-performed the five item modified frailty index in each of these models.

**Table 2:** Adverse Events and Outcomes by MF-6 Category

Patients (% total)	Normal MF-6 n = 6943 (73.4)	MF-6 > 3 n = 2520 (26.6)	Odds Ratio, Interfacility Transfer (95% Confidence Interval)
Mortality^a^	213 (3.1)	183 (7.3)	2.48 (2.02-3.03)
Major adverse event^a^	626 (9.0)	444 (17.6)	2.16 (1.89-2.46)
Infectious complications^a^	457 (6.6)	270 (10.7)	1.70 (1.45-1.99)
Discharge other than home	5,184 (74.7)	2,168 (86.0)	2.09 (1.84-2.37)
Pulmonary embolism^a^	51 (0.7)	17 (0.7)	0.98 (0.85-1.12)
Return to OR^a^	184 (2.8	90 (3.6)	1.36 (1.05-1.76)
DVT requiring therapy	81 (1.2)	31 (1.2)	1.06 (0.70-1.6)
Readmission^a^	467 (6.8)	292 (11.6)	1.82 (1.56-2.12)
LOS (d)	5.7 ± 4.9	7.4 ± 6.5	*P* < 0.001

CI = confidence interval, DVT = deep vein thrombosis, LOS = length of stay, OR = operating room

a *P*<0.01, statistically significant.

## Discussion

Using the ACS-NSQIP database from 2012 to 2019, a six-item modified frailty index predicted short-term adverse events, readmission, and mortality in patients older than 65 years undergoing open reduction and internal fixation of lower extremity, pelvic, and acetabular fractures. We excluded hip fractures for the purpose of this study because we expected the volume of hip fractures would bias this study and that this frail category of patients would have distinct characteristics not applicable to the remainder of the geriatric trauma data set. Compared with patients with an MF-6 score of less than 3, patients with an MF-6 score of three or greater were 209% more likely to have discharge destination other than home, 216% more likely to have major adverse events, and 182% more likely to be readmitted to the hospital.

These results are consistent with previous studies documenting the ability of the five-item modified frailty index to predict poor postoperative outcomes after orthopaedic surgery.^13,24,25,26^ Traven et al^13^ found that the MF-5 was an independent predictor of postoperative complications, readmission, extended hospital stay, and mortality in patients 60 years and older undergoing surgery for hip fractures. Yi et al^24^ found that the MF-5 was a moderate predictor of postoperative complications among patients undergoing surgical fixation of a proximal humerus fracture. Zreik et al^25^ reported that a higher MF-5 score was associated with increased risk of 30-day adverse outcomes, complications, discharge destination other than home, and unplanned readmission after anterior cervical diskectomy and fusion. Wilson et al^26^ found that an MF-5 score of two or greater was associated with increased risk of postoperative complications, hospital readmission, and revision surgery among patients undergoing surgical management of distal radius fractures.

Other systems of quantifying frailty have also been shown to predict risk of adverse events after surgery for fractures of the lower extremity, pelvis, and acetabulum.^22,32,33,34^ Vu et al^33^ found that the 11-question modified frailty index was a notable predictor of mortality, readmission, and complications among patients who received surgery for lower extremity and pelvic fractures. Ondeck et al^22^ evaluated the discriminative ability of several measures of frailty and found the Elixhauser Comorbidity Measure to be the best predictor of adverse events, extended hospital stay, and mortality after hip fracture. Krishnan et al^34^ found that frailty level derived from a comprehensive geriatric assessment was a predictor of mortality and length of inpatient stay among hip fracture patients. Clement et al^32^ found that preinjury independence was a predictor of return to home and mortality in elderly patients with pelvic fractures. Although these systems are useful in predicting outcomes after surgery for the lower extremity, pelvis, and acetabulum, they require more information than the MF-5, and this information is not always easily available to clinicians and researchers.

The MF-6 evaluated in this study supplements the five readily available variables in the MF-5 with one additional variable, hypoalbuminemia, to determine whether this improves predictive ability while still minimizing the number of items on the scale. Existing literature demonstrates the utility of albumin levels in predicting outcomes after orthopaedic surgery.^27,28,29,30^ Kishawi et al^28^ found that patients undergoing total joint arthroplasty with low albumin levels were at markedly increased risk of infection, pneumonia, sepsis, myocardial infarction, and other adverse outcomes compared with patients with normal albumin levels. Wilson et al found that hypoalbuminemia was a predictor of postoperative complications, hospital readmission, revision surgery, and mortality among nongeriatric patients with lower extremity, pelvic, and acetabular fractures. The precise pathophysiology of how hypoalbuminemia increases risk of complications and mortality is unknown, although it may be related to malnutrition or inflammatory states.^35,36^

In addition to supplementing the MF-5 with the hypoalbuminemia variable, this study reports on the most recent possible data from the NSQIP. While previous studies examining the MF-5 have included time frames of 2005 to 2006,^13^ 2005 to 2017,^24^ 2016 to 2018,^25^ and 2007 to 2015,^26^ this study includes NSQIP records from 2012 to 2019. By doing so, this study confirms the utility of the MF-6 in predicting adverse events in the current surgical landscape.

In this study, a receiver-operating curve linear regression analysis of MF-6 scores resulted in an AUC of 0.65 for mortality, 0.62 for major adverse events, and 0.62 for discharge destination other than home. Notably, the AUC for each of these indicators was less than 0.7, which is generally considered to represent good discriminative performance.^37^ This may indicate that additional variables not captured by the MF-6 affect adverse events, readmission, and mortality rates after surgery for lower extremity fractures. The MF-6 is a basic frailty measurement that is easy to calculate but is not able to consider all the nuances of frailty. A formal frailty assessment by a geriatrician likely better captures all the complexities of a particular patient.^38^ Surgical decision making and postoperative management is best done with a collaborative approach between surgeons and geriatricians or internists. However, the MF-6 and similar frailty scales are useful tools to quickly and easily estimate the risk level.

Our findings are similar to AUC values reported by studies of the MF-5 in different patient populations. Zreik et al^25^ reported that for patients undergoing anterior cervical diskectomy and fusion, the MF-5 had an AUC of 0.62 for major complications and 0.65 for discharge destination other than home. Yi et al reported that for patients undergoing surgery for proximal humerus fractures, the MF-5 had an AUC of 0.68 for mortality and an AUC of 0.60 for any adverse event. Notably, these authors found that the American Society of Anesthesiologists score had the best discriminative ability for these patients, with an AUC of 0.75 for mortality and an AUC of 0.65 for any adverse event. These results, combined with our own, indicate that although the MF-6 may not be a perfect predictor of adverse events, it is still a useful tool to assess risk in geriatric patients.

The results of this study must be understood within the context of the following limitations. First, because the ACS-NSQIP only reports on 30 days of follow-up, it was only possible to identify short-term complications and mortality. We were only able to correct for confounders with associated variables in the ACS-NSQIP database. In addition, albumin levels were only recorded for 56% of the patients in the database, potentially resulting in a selection bias. Risk stratification calculators, such as the MF-6, have an inherent limitation in that each variable is given equal weight. As such, a patient with diabetes and hypertension may earn the same score as a patient with congestive heart failure and current pneumonia. Despite these limitations, we were able to demonstrate that the six-item modified frailty index predicts short-term outcomes, complications, and mortality among geriatric patients with lower extremity, pelvic, and acetabular fractures.

## Conclusion

Among patients aged 65 years and older undergoing open reduction and internal fixation for lower extremity, pelvic, and acetabular fractures, the MF-6 score is associated with a 30-day postoperative incidence of major complications, readmission, discharge destination, and mortality. Orthopaedic trauma surgeons should be familiar with frailty in the geriatric population and can use this information in concert with geriatricians or internists to improve perioperative management, tailor discharge planning, and anticipate complications in this patient population. Average patient MF-6 scores can also be used to risk-stratify patient populations more easily as shifts to value-based care continue to develop. Future studies are needed to examine the potential for perioperative interventions and assess longer term outcomes in this patient population using the MF-6 as a screening tool for palliative care consultation and health access expansion.
